# The American Medical Association

**Published:** 1876-06

**Authors:** 


### Editorial.

### The American Medical Association.

In another part of this number we publish a synopsis of the proceedings of the American Medical Association, at its meeting in Philadelphia, for which we are indebted to the *Philadelphia Medical Times.*

Nothing of any special interest or importance, transpired at this meeting beyond the usual routine of business.

The address of the retiring President, Dr. Sims, of New York, was a disappointment and surprise to a large number of the delegates present. While we do not profess to look upon the Code of Ethics as perfect, we should be sorry indeed to see it abolished. It is violated frequently and in other ways than by the proscribing of McMunn's Elixir, Chlorodyne, and other secret remedies. Yet we think that it is a wholesome check to a number of the profession who are not actuated by that "unwritten law" which rules the action of every honorable physician. Dr. Sims tells us that it is violated by not only the rank and file, but by those high in the profession, which strikes us as an argument for increased stringency, rather than abrogation. The matter of a medical man taking out patents upon instruments and appliances which he has originated, is one which has many and strong arguments on both sides; Dr. Sims seems to favor the patenting of all such inventions.

We hope that a full report of the President's remarks will change in some manner their general tone in reference to the Code of Ethics; as they now stand they are unworthy of being pronounced by the head of the American Medical Association, and will receive the sanction of those only who are in favor of fellowship with quacks and impostors and a general "devil take the hindmost" system of practice.

The other topic to which Dr. Sims gave his attention was Syphilis. He does not favor the license and inspection of prostitution as employed in some of the European cities, but proposes to stamp out syphilis by placing its control in the hands of Boards of Health, giving them the same arbitrary power in reference to it as they now have in small-pox and cholera. He would have all immigrants thoroughly examined, and those found tainted should not be allowed to remain in the country. Why limit this proscription to immigrants? If Dr. Sims wishes to keep syphilis out of the country he must also banish those affected with it, who are already here, which scheme would result in a most frightful depopulation of the country. Dr. Sims did well in calling attention to the question, which all admit to be one of great importance, but we fear his plan of meeting the issue is one which will not be found in any measure practicable.

Since writing the above remarks the following from the *Medical Record* has come to hand, which we copy in full:

"THE USE OF PROPRIETARY MEDICINES."

"Considering the great opportunity afforded the late President of the American Medical Association for saying a good word for the profession, we are the more surprised at the manner in which he treated the subject of proprietary medicines in his recent address. No one doubts that physicians have long been prescribing patented medicinal preparations, but such a reprehensible practice cannot be viewed as an argument to prove that part of the Code of Ethics which forbids it should be modified or abolished. The fault of wrong-doing rests not with the law which is intended to prevent it, but with the individual transgressing. As the case now stands, the profession has been humiliated before the world by the virtual admission that not unfrequently it is compelled to call in the aid of quackery to cure disease. If this be a fact, how can we reconcile our pretentious advocacy of the claims of scientific medicine? The use of secret remedies is such a disgraceful reflection upon the resources of our art, that the best we can say for it is that the more the practice is hidden in the humiliation of our own incompetency the better for the profession at large, But what have we to say of the wide-spread publication of an opinion from a leader in the profession, and a president of the representative Medical Association of America, that quack remedies are so useful in the treatment of disease that we cannot do without them, and that in view of making principle consistent with practice, we should alter our Code?

"We cannot believe that the profession will hold itself responsible for this error of judgment on the part of the President of the Association. In the name of that profession we cannot too strongly condemn the principle upon which the argument is founded, and cannot too radically express our mortification of the mistaken policy which gave it such untimely utterance.

"As a question of principle we must take a firm stand against every innovation of quackery, notwithstanding it may be backed by the very priests at our altars. The provisions of the Code in regard to the use of patent nostrums are explicit enough to suit the good intentions of every practitioner, high or low; and as far as the principles are concerned are beyond criticism. If science cannot compete with quackery, humbug, and ignorant impiricism in finding remedies for disease, it is time we enlarged our laboratories, improved our methods of analysis, and studied our materia medicas. What kind of a spectacle do we present to the world in regard to the use of proprietary medicines? We claim, as leaders in professional thought, to be masters of the science of medicine; as well qualified practitioners, to be capable of making every possible application of the resources of that science for the good of our patients; and yet we acknowledge that the veriest quack can blindly stumble upon a remedy which we are glad to use, although ignorant of its composition. We cannot believe that the profession is willing to stultify itself before the public by any such admission. In that view we wish to see the Code stand where it is. The principle upon which it is founded cannot be improved, and we should jealously guard any infringement of its provisions, not only by the ordinary practitioner, but by the President of the Association himself.

"It is our duty and privilege to use anything and everything in the shape of medication which may be of service to our patients. We should fulfil these conditions without recourse to proprietary medicines. Even granting that some quack may throw a valuable remedy in our way, we have the facilities for ascertaining its quality and composition; but failing to do so, we should not, as scientific men, use it at all; in other words, we have no right to prescribe any remedy the composition of which we are ignorant.

"It is often said by physicians in regard to proprietary medicines: 'I know that this compound is a good one, and I use it.' That is well enough as far as it goes, but unless the prescriber takes pains to ascertain upon what the good effects of such a remedy depend, he ignores all the claims of scientific therapeutics and sinks into the hopeless imbecility of aimless empiricism. While we are willing to admit that this evil of striking at disease in the dark is secretly winked at by many physicians, it is nevertheless radically wrong, and should, far from being excused, be unqualifiedly condemned as a disgraceful method of dodging the sacred obligations which we owe to our patients, and of sacrificing the best interests of legitimate medicine.

"As a rebuke to the sentiments of the President on this question, we notice a coincidence almost providential in its significance, in the rejection of one of the papers read before one of the sections of the Association because its author advocated the claims of a secret remedy. A similar action for a similar reason was taken upon a paper read before the State Medical Society at its recent meeting. This is as it should be, and the unanimous vote of the members is a pretty clear indication of the drift of professional opinion. The proper interpretation of the latter will prove to the President of the Association that the profession would much rather raise the standard of professional moral to the high level of the requirements of the Code, than lower that level to suit the requirements of those who are proper subjects for discipline."

